# Association Between the Cytogenetic Profile of Tumor Cells and Response to Preoperative Radiochemotherapy in Locally Advanced Rectal Cancer

**DOI:** 10.1097/MD.0000000000000153

**Published:** 2014-12-05

**Authors:** María González-González, Jacinto Garcia, José A. Alcazar, María L. Gutiérrez, Luis M. Gónzalez, Oscar Bengoechea, María M. Abad, Angel Santos-Briz, Oscar Blanco, Manuela Martín, Ana Rodríguez, Manuel Fuentes, Luis Muñoz-Bellvis, Alberto Orfao, Jose M. Sayagues

**Affiliations:** From the Servicio General de Citometría, Departamento de Medicina and Centro de Investigación del Cáncer (IBMCC-CSIC/USAL), Hospital Universitario de Salamanca-IBSAL, Universidad de Salamanca (MG-G, MLG, MF, AO, MS); Servicio de Cirugía General y Aparato digestivo (JC, JAA, LMG, LM-B); Servicio de Patología, (OB, MMA, AS-B, OB); Servicio de Radio-diagnóstico, Hospital Universitario de Salamanca-IBSAL (MM); and Servicio de Oncología Radioterápica, Hospital Universitario de Salamanca (AR), Salamanca, Spain.

## Abstract

Neoadjuvant radiochemotherapy to locally advanced rectal carcinoma patients has proven efficient in a high percentage of cases. Despite this, some patients show nonresponse or even disease progression. Recent studies suggest that different genetic alterations may be associated with sensitivity versus resistance of rectal cancer tumor cells to neoadjuvant therapy. We investigated the relationship between intratumoral pathways of clonal evolution as assessed by interphase fluorescence in situ hybridization (51 different probes) and response to neoadjuvant radiochemotherapy, evaluated by Dworak criteria in 45 rectal cancer tumors before (n = 45) and after (n = 31) treatment. Losses of chromosomes 1p (44%), 8p (53%), 17p (47%), and 18q (38%) and gains of 1q (49%) and 13q (75%) as well as amplification of 8q (38%) and 20q (47%) chromosomal regions were those specific alterations found at higher frequencies. Significant association (*P* < 0.05) was found between alteration of 1p, 1q, 11p, 12p, and 17p chromosomal regions and degree of response to neoadjuvant therapy. A clear association was observed between cytogenetic profile of the ancestral tumor cell clone and response to radiochemotherapy; cases presenting with del(17p) showed a poor response to neoadjuvant treatment (*P* = 0.03), whereas presence of del(1p) was more frequently observed in responder patients (*P* = 0.0002). Moreover, a significantly higher number of copies of chromosomes 8q (*P* = 0.004), 13q (*P* = 0.003), and 20q (*P* = 0.002) were found after therapy versus paired pretreatment rectal cancer samples. Our results point out the existence of an association between tumor cytogenetics and response to neoadjuvant therapy in locally advanced rectal cancer. Further studies in larger series of patients are necessary to confirm our results.

## Abstract 2

Supplemental Digital Content is available in the text

## INTRODUCTION

Neoadjuvant radiochemotherapy administered prior to surgery to patients with locally advanced rectal carcinomas has proven effective in a substantial percentage of cases^1^; for this purpose, 5-fluorouracile (5-FU) or capacetibine is currently recommended.^1,2^ The beneficial effects of radiochemotherapy include achievement of a lower tumor stage that allows for both less-invasive surgical procedures and preservation of the sphincters, and at the same time it is associated with less clinical complications after surgery.^3^ This also has been shown to lead to a reduced risk of relapse and a better patient outcome (eg, improved overall survival)^2,4,5^ as well as to an improved quality of life. Despite this, response to neoadjunvant treatment remains highly variable, ranging from complete histopathological response to absence of response, and even tumor progression in a minority of cases.^6^ At present, there is no consensus method about how to evaluate response to neoadjuvant treatment; however, the TNM staging and the Dworak regression system are well-accepted approaches, which are most commonly used to evaluate response to radiochemotherapy prior to surgery.^6,7^

In recent years, controversial results have been reported in the literature as regards the most informative predictors for response to neoadjunvant therapy.^8^ Thus, expression of specific molecules evaluated by immunohistochemical methods, such as p53, has shown discrepant results.^8,9^ In turn, preliminary reports have also found an association between specific genetic/chromosomal alterations and response of locally advanced rectal carcinomas to neoadjuvant therapy. Thus, Grade et al^10^ found a greater frequency of gains of the 7q32-q36 and 7q11-q31 chromosomal regions and amplification of chromosome 20q11-q13 as assessed by comparative genomic hybridization (CGH-arrays), among 21 responder patients out of 42 cases studied; in this report, response was determined by tumor downstage after radiochemotherapy. Based on the same methodology applied to a series of 48 patients, Molinari et al^11^ identified a large number of chromosomal alterations that could be useful to discriminate between responder (44%) and nonresponder (56%) patients, as evaluated by the Dworak criteria. However, these findings have not been prospectively validated in a larger cohort of patients using the same (eg, histopathological) treatment response criteria. In turn, none of the techniques that have been applied so far for the genetic/genomic characterization of responder versus nonresponder cases (eg, CGH-arrays) has provided information about the clonal heterogeneity of rectal cancer at the intratumoral single-cell level. This could be particularly relevant when different clones coexist at variable frequencies in a tumor sample, and only part of such clones is potentially involved in tumor sensitiveness versus resistance to radiochemotherapy administered prior to surgery.^12^

In the present study, we applied multicolor interphase fluorescence in situ hybridization (*i*FISH) for the analysis of 51 different DNA sequences distributed across those chromosomes and chromosomal regions most frequently altered in locally advanced rectal carcinomas, in a series of 45 consecutive patients in which paired pretreatment and posttreatment tissue biopsies were obtained and studied. Our major goal was to establish the specific pathways of clonal evolution inside individual tumors and to investigate their potential association with response versus resistance to radiochemotherapy administrated prior to surgery, as assessed by the Dworak regression system.^7^

## METHODS

### Patients and Samples

A total of 45 patients (15 women and 30 men; median age of 67 years, range 39–85 years) diagnosed of locally advanced rectal cancer at the University Hospital of Salamanca (Salamanca, Spain) between September 2007 and May 2011 were included in this study. Before treatment was given, patients were grouped according to the *u*TNM classification using imaging techniques, for example, rigid rectoscopy endorectal ultrasound, colonoscopy, computed tomography, and magnetic resonance imaging. The most relevant clinical and laboratory data about the patients are summarized in Table 1 and described in more detail in Supplementary Table 1, Supplemental Digital Content 1, http://links.lww.com/MD/A75. In every case, radiochemotherapy consisting of long-course radiotherapy with 50.4 Gy administrated in 25 to 28 fractions, plus capetacitabine (800–825 mg/m^2^), were given prior to surgical removal of the tumor. At this latter time point, the degree of response was scored from grade 0 (absence of tumor regression) to grade 4 (complete tumor regression), following the Dworak system (Table 1).

**TABLE 1:**
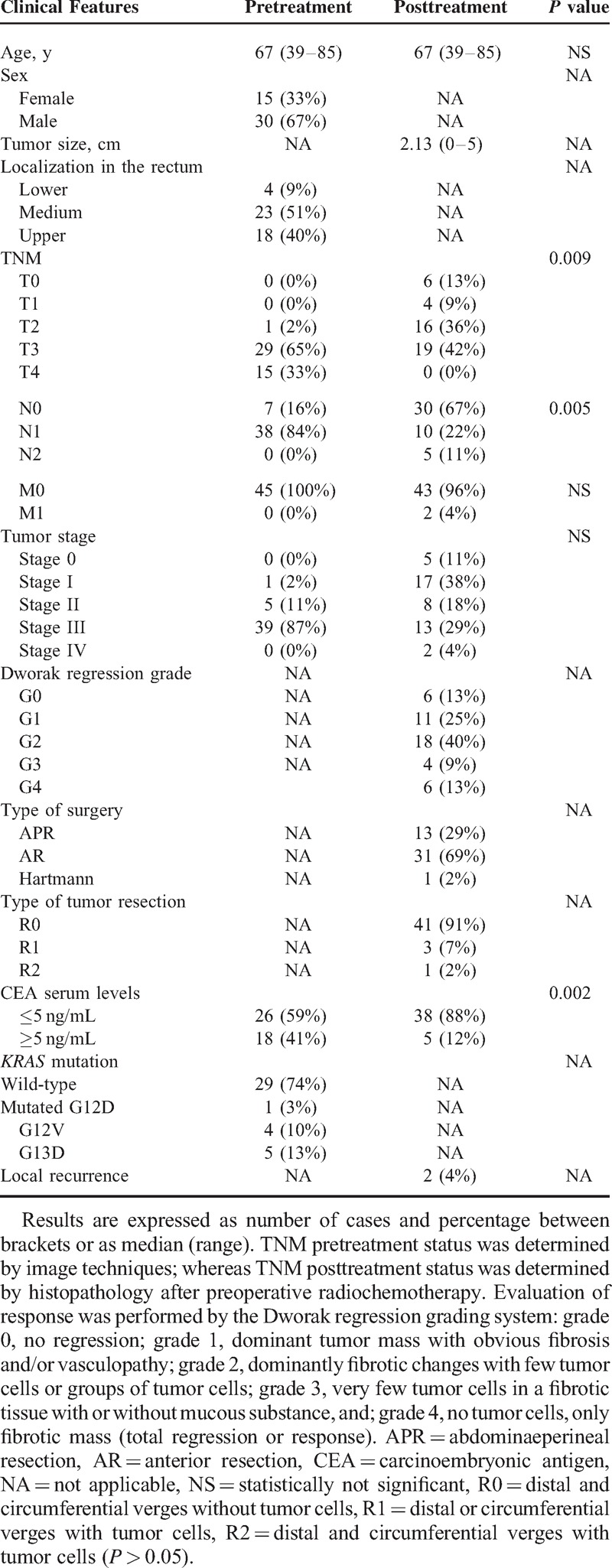
Clinical and Biological Characteristics of Locally Advanced Rectal Cancer Patients (n = 45) Before and After Treatment (radiochemotherapy) Given Prior to Surgery

Overall, 76 tissue samples were analyzed by *i*FISH; these included 45 pretreatment tissue biopsy samples and 31 (paired) posttreatment samples. Those 14 cases with unpaired follow-up samples corresponded to 6 cases showing complete regression of the tumor after radiochemotherapy plus 8 patients who had no left-over tissue material after the required diagnostic procedures. All samples were sequentially fixed, stained with hematoxylin and eosin and microscopically evaluated to confirm the presence of tumor cells and to assess the quality of the samples to be used for *i*FISH analyses. The study was approved by the Local Ethics Committee of the University Hospital of Salamanca (Salamanca, Spain).

### *i*FISH Assays

Premixed, methanol/acetic (3/1 vol/vol) fixed, single-cell suspensions from each individual biopsy tumor sample obtained either pre- or posttherapy (n = 76) were used for *i*FISH studies. A set of 51 different probes specific for those chromosomal regions most frequently altered in rectal carcinomas was systematically applied in triple stainings for the analysis of each individual sample; see Supplementary Table 2, Supplemental Digital Content 2, http://links.lww.com/MD/A75, which illustrates the fluorochrome-labeled interphase FISH probes used for the cytogenetic characterization of locally advanced rectal carcinomas. To precisely define the specific pattern of chromosomal alterations coexisting in individual tumor cell clones within a sample, further appropriate multicolor stainings were performed, whenever necessary. The methods and procedures used for the *i*FISH studies have been previously described in detail elsewhere.^13^

### Statistical Methods

For all continuous variables, mean values and their standard deviation (SD) and range were calculated; for dichotomic variables, frequencies were reported (SPSS software 15.0 package; SPSS Inc, Chicago, IL). To evaluate the statistical significance of differences observed between groups, the Student *t* and the Mann–Whitney *U* tests were used for continuous variables as well as Wilcoxon test to paired groups, depending on whether they displayed or not a normal distribution, respectively (SPSS); for qualitative variables, the *χ*^2^ test was applied and McNemar test to paired groups (cross-tab; SPSS). Statistical significance was set at a *P* value of <0.05.

## RESULTS

### Distribution of Chromosomal Alterations in Locally Advanced Rectal Cancer Before and After Radiochemotherapy

**FIGURE 1:**
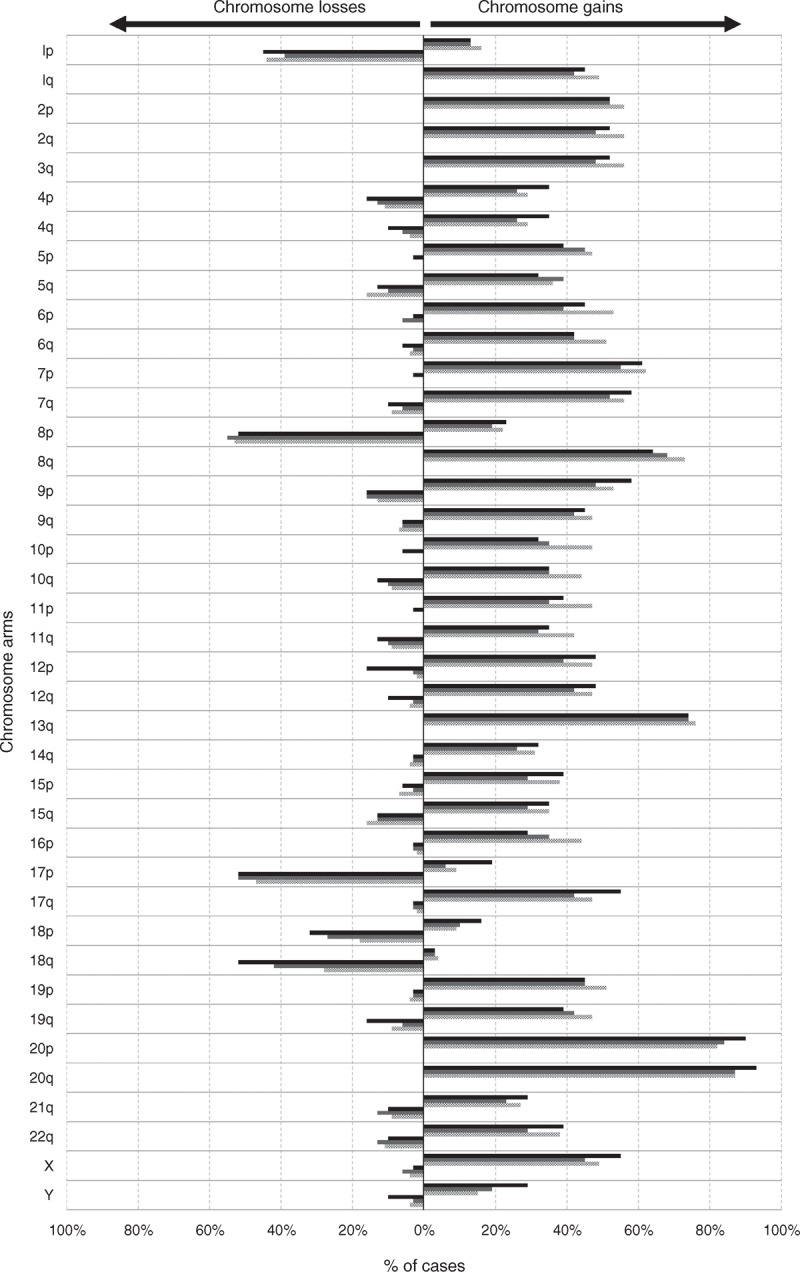
Frequency of chromosome gains and losses identified by interphase fluorescence in situ hybridization in locally advanced rectal carcinoma samples obtained prior to therapy (dotted bars; n = 45 and grey bars; n = 31 paired samples to those studied after therapy) and after radiochemotherapy (dark bars; n = 31). Those chromosomal regions most frequently showing recurrent losses and gains were localized in chromosomes 1p, 8p, 17p, and 18q, and the 8q, 13q, and 20q chromosomal regions, respectively.

For all chromosomes analyzed, most of the rectal cancer samples obtained before treatment (44/45 tumors) showed complex karyotypes with numerical and/or structural abnormalities involving ≥2 chromosomal regions; the remaining case showed no chromosomal alterations for the 51 different probes investigated. Overall, gains of chromosomal regions were more frequently detected than chromosome losses (44% versus 9%, respectively; *P* < .001) (Figure 1). In most instances, chromosomal gains reflected underlying polyploid karyotypes, being polysomies of chromosomes 2 (58% of tumors), 3 (56%), 6 (47%), 7 (56%), 12 (47%), 13 (75%) and 20 (87%) the individual numerical chromosomal alterations more frequently detected (Figure 1). In turn, the most frequent structural chromosomal alterations corresponded to losses of the 1p (44% of tumors), 8p (53%), 17p (47%), and 18q (38%) chromosomal regions and to gains of the 1q (49%) and 13q (75%) chromosomal regions, in addition to amplification of the 8q (38%) and 20q (47%) chromosomal regions. Of note, no significant associations were found between alterations of individual chromosomes and clinical disease features such as age and tumor localization (*P* > 0.05).

Posttreatment rectal cancer samples (n = 31) from surgical specimens obtained after a median of 5 months from diagnosis (range 3–7 months) showed a similar (eg, related) cytogenetic profile to that found in their paired pretreatment tumor samples (Figure 1). Despite this, significant differences in the number of copies detected for chromosomes 8q (*p* = .004), 13q (*P* = .003), and 20q (*P* = .002) were found between pretreatment rectal cancer samples and their paired posttreatment samples, see Supplementary Figure 1, Supplemental Digital Content 3, http://links.lww.com/MD/A75, which illustrates the numerical alterations of chromosomes 8q24 (panel A), 13q34 (panel B), and 20q13 (panel C) in paired pre- and posttreatment tumor samples from locally advanced rectal cancer patients (n = 31). Notched-boxes extend from the 25th to 75th percentile values; the lines in the middle and vertical lines correspond to median values and the 10th and 90th percentiles, respectively. In addition, a slightly lower (*P* > 0.05) frequency of losses of the 1p (39% vs 45%, respectively), 18p (23% vs 35%), 18q (42% vs 55%), and 19q chromosomal regions (6% vs 16%), as well as of gains of the 7p (55% vs 61%) and 17q chromosomal regions (42% vs 55%) and of chromosome Y (16% vs 28%) in males were found before versus after radiochemotherapy (Figure 1).

### Chromosomal Alterations and Local Response to Preoperative Radiochemotherapy

Upon grouping rectal cancer patients according to tumor response to radiochemotherapy administered prior to surgery, a significant association was found between response to radiochemotherapy (as assessed by the Dworak grade) and rectal carcinomas displaying alterations of chromosomes 1p (*P* = 0.0002), 1q (*P* = 0.03), 11p (*P* =  0 .04), 12p (*P* = 0.04), and 17p (*P* = 0.03) (Table 2); in contrast, no significant differences were found for none of the other chromosomes analyzed; see Supplementary Table 3, Supplemental Digital Content 4, http://links.lww.com/MD/A75, which illustrates the chromosomal alterations detected at diagnosis in locally advanced rectal cancer tumors (n = 45) grouped according to response to radiochemotherapy administered prior to surgery (Dworak regression grades). Therefore, del(17p) and polysomies of chromosomes 1q, 11p, and 12p were significantly more frequent among nonresponder versus responder patients; in contrast, del(1p) was found in a higher percentage of responder versus nonresponder patients (Table 2).

**TABLE 2:**
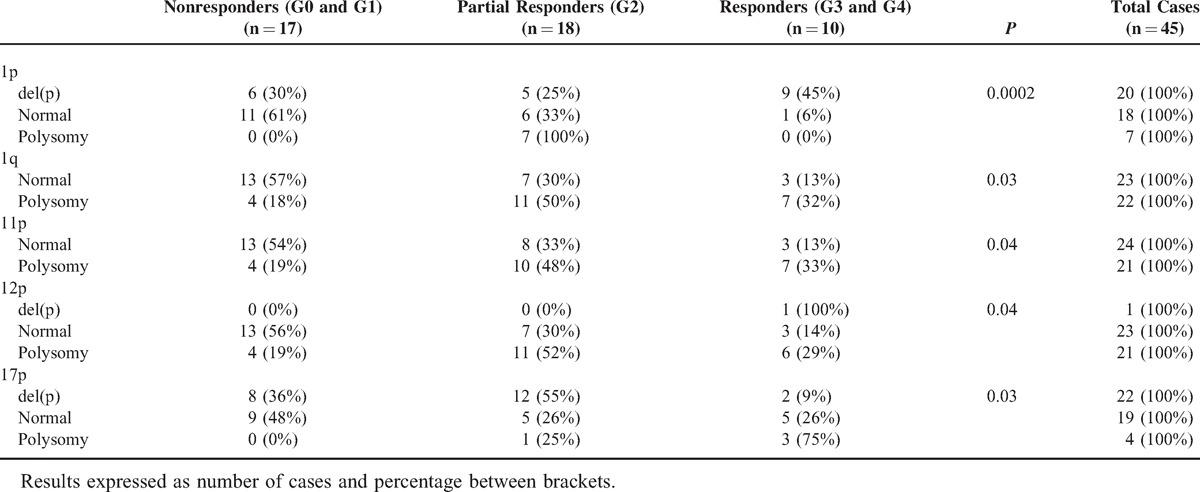
Chromosomal Alterations Detected at Diagnosis in Locally Advanced Rectal Cancer Tumors (n = 45), Which Were Associated With the Grade of Tumor Regression (Dworak Grade) After Radiochemotherapy was Administrated Prior to Surgery

### Intratumoral Patterns of Clonal Evolution and Response to Therapy Prior to Surgery

Detailed analysis of the pattern of chromosomal alterations of single tumor cell nuclei within individual tumors revealed the presence of ≥2 distinct tumor cell clones in 31 of 45 cases. Those clones, which contained chromosomal alterations common to all tumor cells in an individual tumor, were considered to be ancestral tumor cell clones, whereas those presenting alterations, which involved only a fraction (>10%–90%) of all tumor cells, were considered to be secondary clones.^13,14^ Ancestral tumor cell clones were highly variable, but they commonly showed recurrent loss of chromosome 8p (51% of cases) and gains of the 8q, 13q, and 20q chromosomal regions (60% of cases).

**FIGURE 2:**
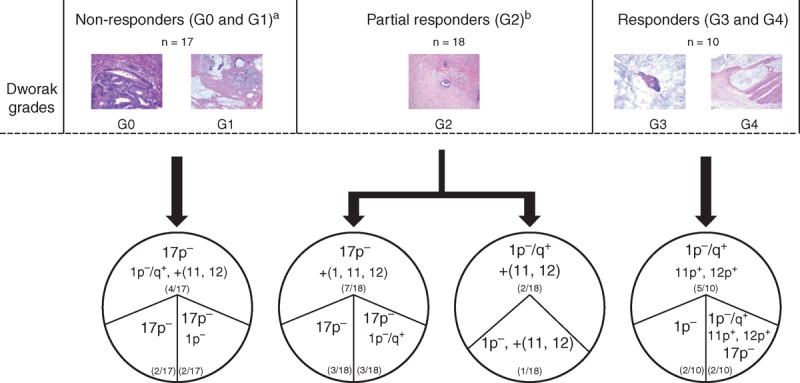
Intratumoral cytogenetic heterogeneity of locally advanced rectal cancer prior to radiochemotherapy as evaluated by the cytogenetic profile of the ancestral tumor cell clones grouped according to response to therapy (Dworak grade). (A) 9/17 non-responder cases (G0 and G1) showed other heterogeneous cytogenetic profiles in their ancestral tumor clones, which are not represented here; (B) 5/18 partial responder (G2) cases also showed other cytogenetic profiles in their ancestral tumor clones, which are not represented here.

**FIGURE 3:**
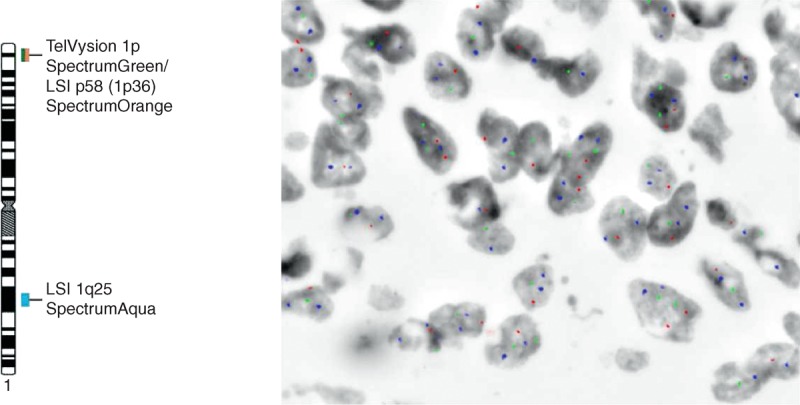
Interphase nuclei from a biopsy sample of a patient with locally advanced rectal cancer who achieved complete tumor regression after neoadjuvant therapy. Cell nuclei shows loss of the 1p chromosomal region, as defined by simultaneous hybridization for the Tel1p (green spots), 1p36 (red spots), and 1q25 (blue spots) chromosome 1 regions; altered nuclei only show one copy for the Tel1p and 1p36 probes and 2 copies for the chromosome 1q25 probe.

Most interestingly, a clear association was found (*P* < .05) between those cytogenetic profiles of the ancestral tumor clone detected prior to surgery that involved alterations of chromosomes 1p, 1q, 11p, 12p, and 17p and response to radiochemotherapy (Figure 2). Thus, ancestral tumor clones carrying del(17p) alone (2/17 cases; 12%) or in combination with either del(1p) alone (2/17 cases; 12%) or in association with gains of chromosomes 11 and 12 (4/17 cases; 23%, were typically found among non-responder (G0/G1) cases, 8/17 cases (47%) vs. 2/10 (20%) responder cases (G3/G4). Similarly, del(17p) alone (3/18; 17%) or in association with either gains of chromosomes 1, 11, and 12 (7/18; 39%) or structural alterations of chromosome 1, for example, del(1p), and gain of chromosome 1q (2/18; 11%) was also present in the majority of the partial responder (G2) cases (12/18 cases; 75%), whereas absent in most (8/10 [80%]) responder cases (*P* = .04). Partial responders also recurrently showed del(1p) (3/18 cases; 17%) in their ancestral clone in association with gains of chromosomes 11 and 12 alone (1/18 cases) or in combination with +1q (2/18 cases). Interestingly, similar profiles characterized by del(1p) were present in the ancestral tumor cell clone of all but one responder (G3 and G4) cases (9/10; 90%) as the earliest chromosomal alteration (Figure 3); in such cases, del(1p) was detected as the only chromosomal alteration (2/10; 20%) or it was associated with gains of chromosomes 1q, 11p and 12p (5/10; 50%) or del(17p) in 2/10 cases (20%).

Of note, secondary chromosomal alterations observed in responder cases (eg, 8q, 13q, and 20q amplifications) were similar to those identified also in secondary clones from patients with a poorer response, but they were detected in higher (*P* = .002) percentages of tumor cells in posttreatment samples; see Supplementary Figure 2, Supplemental Digital Content 5, http://links.lww.com/MD/A75, which illustrates the genetic heterogeneity of locally advanced rectal carcinomas: hypothetical intratumoral aneuploidization pathways deduced for those chromosomal alterations (1p, 1q, 11p, 12p, and 17p) which showed a significant association with response to therapy as assessed by the Dworak grading system. Overall, the cytogenetic patterns associated with clonal evolution of the ancestral tumor cell clones detected in the tumor samples studied prior to radiochemotherapy versus those observed in posttreatment samples were variable, but they frequently involved gradual copy number gains of chromosomes 8q, 13q, and 20q; see Supplementary Figure 2, Supplemental Digital Content 5, http://links.lww.com/MD/A75, which illustrates the genetic heterogeneity of locally advanced rectal carcinomas: hypothetical intratumoral aneuploidization pathways deduced for those chromosomal alterations (1p, 1q, 11p, 12p and 17p), which showed a significant association with response to therapy as assessed by the Dworak grading system.

## DISCUSSION

In recent years, administration of neoadjuvant radiochemotherapy prior to surgery, to locally advanced rectal cancer patients has become standard clinical practice.^1^ Despite such treatment strategy has been associated with an overall benefit for the patient,^2,3,15^ still the degree of response to radiochemotherapy remains highly variable among different patients.^16^ Thus, although 5% to 25% of patients achieve complete remission (complete absence of tumor cells) and 40% to 60% reach a significant reduction in tumor mass, around 20% to 30% of cases do not respond to therapy and some of them may even show disease progression.^16^ At present, it still remains poorly understood which tumors are more prone to be sensitive versus resistant to radiochemotherapy administered prior to surgery and which are those factors that determine a good versus poor response to preoperative radiochemotherapy. Among other variables, the cytogenetic background of tumor cells has been suggested to potentially play a role, due to the relatively high cytogenetic heterogeneity of tumor cells among different tumors, as well as within individual tumors.^13,17,18^ Here we used multicolor *i*FISH for detailed analysis of the cytogenetic heterogeneity of locally advanced rectal cancer tumors, both at the inter- and the intratumoral cell levels, evaluated before and after preoperative radiochemotherapy; our major goal was to gain insight into the most frequent pathways of intratumoral clonal evolution that could be associated with response versus resistance to neoadjuvant therapy.

Previous studies have consistently identified a high frequency of complex karyotypes with gains of chromosomes 7, 8q, 13q, and 20 and losses of the 1p, 5q, 8p, 14q, 15q, 17p, and 18q chromosomal regions,^10,11,13,19–21^ among locally advanced rectal cancer tumor patients. In line with these observations, all, except 1, rectal cancer tumor samples obtained prior to therapy showed complex karyotypes with ≥2 altered chromosomes. As previously described, the most frequent alterations here observed included gains of chromosomes 7, 8q, 13q, and 20q and losses of the 1p, 8p, 17p, and 18q chromosomal regions. Of note, alterations of the 8q, 13q, and 20q chromosomal regions were observed at similar frequencies in all groups of patients defined according to response to therapy (eg, Dworak grades). Interestingly, however, important differences among cases showing a different grade of response to neoadjuvant therapy were identified as regards the patterns of intratumoural clonal evolution, particularly the cytogenetic profiles of the ancestral tumor cell clones for chromosomes 1, 11p, 12p, and 17p. Thus, del(17p) predominated among the ancestral clone of nonresponder patients (Dworak grades 0 and 1), whereas alterations of chromosome 1 in the absence of del(17p) were more frequently observed in the ancestral tumor cell clone of responder (Dworak grades 3 and 4) cases. Partial responders (Dworak grade 2) included a heterogeneous group of patients from both the histopathological and the genetic point of view with cases carrying either alterations of chromosome 1 (as the responder G3 and G4 cases) and/or displaying del(17p) in their ancestral tumor cell clones (similarly to the nonresponder G0 and G1 cases). Altogether, these results suggest that response to radiochemotherapy administrated prior to surgery is associated with specific cytogenetic profiles reflected by potential “driver” chromosomal alterations, further studies being necessary to investigate the precise molecular mechanisms involved in tumor cell sensitivity and resistance to therapy.

Despite all the above, combined loss of chromosomes 1p and 19q is well documented to be associated with sensitiveness to radiochemotherapy in oligodendroglial tumors,^22,23^ the *PRDX1*antioxidant protective gene encoded at chromosome 1p34 being potentially involved in radiochemosensitivity in these tumors.^22^ In addition, gains of chromosome 1q^24^ have also been reported to be associated with sensitivity to chemotherapy in glioma patients^24^ and similarly, gain of the *ABL2* gene encoded at chromosome 1q25 in non-small cell lung cancer has been related to a good in vitro response to chemotherapy as well.^25^ In contrast, del(17p) has long been reported in metastatic colorectal carcinomas where it has been associated with a poorer outcome.^20,21,26^ Furthermore, del(17p) is frequently associated with *TP53* mutations localized at the deleted region in the retained 17p13 chromosomal band, and *TP53* mutations have long been associated with a poor response to radiochemotherapy of both colorectal cancer^27,28^ and other cancer types, (eg, head and neck carcinomas treated with 5-FU).^29^ In addition, del(17p) alone or in combination with TP53 mutations has also proven to be associated with resistance to chemotherapy in patients with chronic lymphocytic leukemia.^30,31^ In line with these findings in a meta-analysis, Chen et al^32^ have recently shown that the *TP53* status could be used as a predictive biomarker for rectal cancer patients treated with radiochemotherapy prior to surgery. For decades now, it is well established that mutated *TP53* frequently prevents tumor cells from undergoing apoptosis, even in the presence of marked DNA damage^33,34^; consequently, this may contribute to explain not only the lower response here observed to radiochemotherapy among cases which carry del(17p) in their ancestral tumor clone, but also the occurrence of both cytogenetic and clinical progression after therapy in a subset of these cases. In line with these findings, Petty et al^35^ have also identified the expression of the *APRIL* gene, a paracrine/autocrine molecule involved in signaling for cell proliferation, which is encoded in the vicinity of gene *TP53*, to be significantly associated with resistance to 5-FU in colorectal cancer patients. Altogether, these findings are in line with our observations pointing out the overall association between presence of del(17p) and a poorer response to radiochemotherapy.

Regarding chromosome 12p, Chen et al^36^ have reported an association between del(12p) and complete response to neoadjuvant treatment of rectal cancer patients. However, it should be noted that in our series, gains of chromosome 12p were more frequently detected than del(12p), the only case that displayed del(12p) corresponding to a responder patient (G3). Despite this, the precise mechanisms involved in the association here described between the gains of chromosomes 12p and 11p, in association with del(1p), and response to therapy remain to be elucidated.

Interestingly, similar cytogenetic profiles were found in our series between paired pre- and posttreatment tumor samples, although the frequency of individual chromosomal alterations was typically slightly increased after therapy. Moreover, in most cases, the predominant tumor cell clone detected before therapy was also highly represented in posttreatment samples; however, in a subset of our patients (n = 7), minor clones detected prior to radiochemotherapy became dominant in posttreatment samples. Overall, the cytogenetic relationship here observed between pre- and posttreatment samples supports previous observations made by others in different cancer types such as ovarian cancer,^37^ central nervous system tumors,^38^ hematological malignancies,^39,40^ or cervical cancer.^41^ Acquisition of new (additional) genetic alterations and clonal selection has also been recurrently described for different cancer types.^17,18,42^ However, we cannot fully rule out that in these latter cases, variations in clone size are due to a different distribution of distinct tumor cell clones in different areas of the tumor.^17,18,42,43^ Of note, among other chromosomal alterations, posttreatment samples frequently carried additional gains of the 8q, 13q, and 20q chromosomal regions, independently of the degree of response to adjuvant radiochemotherapy. Interestingly, all 3 chromosomal alterations have been recurrently associated with tumor progression and more aggressive phenotypes.^19,20,44^ The exact meaning of the acquisition of multiple/additional copies of these chromosomal regions remains to be elucidated.

In summary, in the present study, we observed significant association between the cytogenetic profile of the ancestral tumor cell clones of locally advanced rectal cancer patients and response to radiochemotherapy administered prior to surgery: del (17p) was associated with poor-responders, whereas del(1p) was more closely associated with a better response. Further studies are required to confirm our results and to determine the precise molecular mechanisms involved in such association and discover potential ways to reverse them.
